# Managing Children's Anxiety During COVID-19 Pandemic: Strategies for Providers and Caregivers

**DOI:** 10.3389/fpsyt.2020.552823

**Published:** 2020-11-12

**Authors:** Ying Qi Kang, Tammy S. H. Lim, Elizabeth Sarah Ragen, Mae Yue Tan, Ramkumar Aishworiya

**Affiliations:** ^1^Department of Paediatrics, Yong Loo Lin School of Medicine, National University of Singapore, Singapore, Singapore; ^2^Khoo Teck Puat – National University Children's Medical Institute, National University Health System, Singapore, Singapore

**Keywords:** anxiety, COVID-19, children, caregivers, pandemic

## Abstract

The Coronavirus Disease 2019 pandemic by virtue of its sudden, unprecedented and widespread nature, has led to a multitude of psychological effects on individuals across societies. This includes anxiety which has important implications on the daily functioning, physical and mental health of individuals. Children are a vulnerable group of the population who can experience anxiety which potentially can lead to long-lasting implications on their health for years to come. It is thus important that their caregivers, including parents and healthcare professionals be aware of strategies that can help with anxiety in children. This article discusses anxiety in children in the context of the COVID-19 pandemic and outlines strategies that may be helpful.

## Introduction

The Coronavirus Disease 2019 (COVID-19) pandemic has had multiple implications on individuals worldwide (1). Society members are facing unprecedented changes to their usual routines as governments implement measures to mitigate the spread of the pandemic. Examples include closure of schools with children doing home-based learning (2) in many countries including Singapore and parents working from home as part of social distancing regulations. These measures inevitably lead to changes in the family structure and routine for children (3, 4). Furthermore, as part of the pandemic response, there are also restrictions on regular psychological support systems, such as meeting with friends, physical exercise, and religious activities. The combination of risk factors such as the multitude of change in daily lives, information overload about the pandemic, fear of the unknown, coupled with the reduction in psychological support, can result in heightened anxiety in adults and children alike (5). This has also been described in research on previous epidemics (6, 7).

While worries and fears are a natural and adaptive part of development, when these feelings are persistent and excessive, they can cause distress and significant impairment of an individual's daily functioning (8). It is thus important to identify and address anxiety promptly. In particular, it is important to address anxiety in children. According to the American Psychological Association (APA), anxiety disorders are the most prevalent of all mental health disorders that present in children and youth (9). Anxiety in children can often be harder to detect as children are still maturing cognitively and thus manifest anxiety differently from adults. Symptoms of anxiety manifest differently in different children (10). Some children may present with physical symptoms such as nausea, or stomach aches (11). They may also appear distracted or more inattentive than usual, have increased separation anxiety from caregivers and may have changes in their appetites, feeding and sleeping habits. Some may express their anxiety verbally if they are able to do so. They may also do so through new internalizing behaviors such as being more withdrawn and quiet or by externalizing behaviors such as tantrums and challenging behaviors (12). Healthcare professionals may come across children with anxiety in various settings. This article outlines four main strategies that healthcare professionals can share with families to help children manage their anxiety and feel safe during this turbulent period: Acknowledge, Discuss, Do, and Reflect.

### Acknowledge: Acknowledge Change

The simple step of recognizing and being aware of the changes that have occurred can help children feel that their concerns are validated (13). Often, caregivers may attempt to comfort their child with statements such as “there's no need for you to worry.” These are well-intentioned, but may actually appear to dismiss the child's concerns (14). With younger children, it might be helpful to create a visual list of what has changed and what has not changed in their lives. This could help children feel less alone in their feelings when they know that the changes that they perceive are recognized by another person as well. Further, not only should caregivers acknowledge the changes, but they should recognize that these changes can lead to fears and possibly anxiety in children. This will then prompt caregivers to look out for potential behavioral manifestations of anxiety, and address it earlier. When their caregivers acknowledge the change and its possible consequences, children will in turn be aware that they can go to their caregivers for support.

Asking children specifically to express their feelings and thoughts is another strategy that is helpful. This is because how a child perceives the morbidity and mortality related to COVID-19 and the range of mitigating measures to curb its spread can lead to feelings of fear, confusion, anger and even guilt. Hence, providing regular opportunities for them to express themselves will be an opportunity to better address underlying fear and anxiety. This can be done through verbal expression (for example, initiating a conversation), writing (for example, journal entries), art and play- depending on the child's developmental age and cognition. It is important not to dismiss their feelings or react negatively to their anxiety. Apart from facilitating acknowledgment and validation of their feelings, their perspectives can also be gently corrected where required to reduce anxiety (15).

### Discuss: Provide an Avenue to Discuss the Situation and the Child's Feelings

Providing children with accurate information from reliable sources tailored to their age and level of understanding will help them understand the pandemic better. It is vital that children be able to trust their caregivers to be a reliable source of information as this will help to assuage their anxiety. Where appropriate, update children as the situation evolves so that they can better grasp the need for the constant changes around them. Having an accurate understanding of the situation, can be empowering, and it can help them to better regulate their feelings of anxiety.

Discussing with children the rationale behind the changes implemented and the positive aspects of the situation can be helpful as well. Framing the measures in the light of public safety, i.e., to keep everyone safe, is an example of shifting the child's focus to a positive angle. Common terms that are now associated with the pandemic can be used as both a teaching tool and as a means of assurance. For example, the term “essential services” can be a discussion point about the people who are an integral part of society and are crucial to the fight against COVID-19. This can also help assure children of the people and steps being taken to safeguard the public. Understanding that there are reliable systems, resources and people (e.g., healthcare workers, essential workers, scientists, government officials, etc.) dedicated to managing the pandemic can reduce the sense of helplessness and anxiety levels of children. Caregivers can also talk to their child about the specific steps taken by the family and school to deal with the pandemic.

While discussing the pandemic with children, it is important to be mindful of one's own emotions and thoughts (16). The caregiver's emotions and behaviors influence the child's response. Caregiver/parental co-regulation, through scaffolding of the child's emotions and with strategies to help the child regulate emotions, is crucial in helping the child develop emotional regulation as an individual (17, 18). In addition, caregivers should refrain from discussing their own concerns about the pandemic (for example, financial or employment concerns) and avoid having arguments around the child as this can lead to heightened anxiety in him/her. While not all children can understand or discuss the information and their own feelings, they can pick up on cues from the adults around them. Caregivers should also be aware of their tone and choice of words. Use a calm and matter of fact tone. Extreme statements meant to induce fear and compliance to instructions should be avoided (19). For example, a sweeping statement such as “If you go out to play, you will get sick with COVID-19” will not be helpful. These can inadvertently increase the child's paranoia around this pandemic.

### Do: Maintain Routines and Empower the Child

Predictability is very important for an anxious child as it establishes a sense of stability which can help him/her to feel safe (20). Caregivers can actively plan to maintain consistency in the child's environment and follow a daily routine especially during this period. Anchor a child's routine in daily activities like mealtimes, sleep, and family time and inform the child about his/her schedule. A routine can also distract the child from anxiety-provoking thoughts and keep him/her focused on the present (21). Families should continue to have bonding time, and discussions should not revolve solely on COVID-19 content. This can be an opportunity for families to be more deliberate about incorporating dedicated bonding time through the week; families may learn new skills, develop new routines and traditions together as a family unit. A strong and intact family unit can increase one's resilience in weathering the storm and stressors of the pandemic (22).

Giving children some degree of control over their daily choices can be helpful as anxiety can be reduced when a child feels in control (23). Whenever there is an opportunity, allow a child to make choices about daily matters (for example, a meal choice). Directing children to focus on activities that they can do themselves, for example, practicing good hand hygiene, can empower them as well. Focus on what the child can control, and tangible actions that the child can perform such as having healthy meals, adequate sleep and regular exercise rather than worrying about a situation that is not within their control. Including even short durations of physical activity as part of the daily routine has been found to be helpful in alleviating anxiety (24).

Equipping children with coping strategies is important. Different children also react differently to fears, especially when they become overwhelming. For the child who worries constantly and is unable to focus on the task at hand, dedicated “worry time” can be useful- set aside 10 to 15 min each day to allow a child to talk or write about his/her worries. During this time, be a good listener and allow the child to have uninterrupted time to express themselves. However, once this dedicated time is over, the child is firmly encouraged to no longer focus on their anxieties and do other tasks. For the child who is withdrawn and hard to engage, provide outlets for them to express their feelings, for example, through writing and drawing. Caregivers can then start the conversation by asking them about what they wrote or drew. Children who internalize their worries may also benefit from talking about it from a 3rd person's point of view. For example, they can pretend that it's their toy that is feeling worried and the parent and child can then have a chat with the toy about its feelings. Further, some physical methods of coping with anxiety include deep breathing exercises and progressive muscle relaxation, which can be practiced jointly by the caregiver and the child (25).

Technology and social media is a double-edged sword. Excessive exposure to COVID-19 related news can result in greater anxiety, hence caregivers should limit the child's exposure. Specific times of the day could be set aside for watching and discussing such news. Monitoring the content that children are exposed to through various media is essential (26). Graphic images and threatening content on media can result in long lasting fears in children (27). On the other hand, social media can be used to help children (and adults) maintain their social networks. For example, children can have video calls with their grandparents who are unable to physically visit, have “play dates” or engage in activities with their friends/schoolmates, or keep in touch with their teachers over social media platforms with caregiver supervision.

### Reflect: Self-Care and Caregiver Wellness

Unchecked parental anxiety can result in higher anxiety levels in the child and make them feel unsettled (28). As the child's feelings can be influenced by the caregiver's own emotions (16), caregivers should regularly reflect on their own feelings and be mindful not to project this on their children. Parents of children with chronic illness have higher anxiety levels than parents of healthy children (29). Predictors of anxiety in these caregivers include a heavy caregiver burden, poor emotional well-being, low self-esteem and a negative coping style (30). It is not unexpected that given the pandemic situation, caregivers may feel even higher levels of anxiety. Hence it is essential that the caregivers actively set aside time for their own mental and emotional well-being, for example, to unwind or pursue their own hobbies. It is important for caregivers to take care of themselves so that they can better care for their children. They should also seek professional help if they are facing significant strain and anxiety at home for any reason. By demonstrating active seeking of self-care, parents act as positive role models for children in coping with anxiety.

## Discussion

In conclusion, the current pandemic may lead to many children having heightened levels of anxiety. As healthcare professionals, we encounter children in many settings and have an opportunity to help their caregivers positively. Fortunately, children are resilient. They can adjust and adapt to new situations quickly; this is especially so if they have secure attachments and a responsive relationship with a caregiver (31). We hope that these strategies can empower healthcare professionals to support children and their families as they navigate this COVID-19 pandemic. Teachers and educators, and other individuals who work closely with children, may likewise find these strategies useful. It is also prudent for healthcare professionals to have heightened vigilance for anxiety in children, especially in those with a known medical history of developmental disabilities and chronic medical illnesses. Beyond these strategies, if parents are concerned, they should be encouraged to seek professional help early to manage their child's anxiety before it causes functional impairment. Likewise, parents should also be encouraged to seek professional help for themselves if they feel overwhelmed.

## Data Availability Statement

The original contributions presented in the study are included in the article/supplementary material, further inquiries can be directed to the corresponding author/s.

## Author Contributions

YK and RA conceptualized and wrote the manuscript. TL, MT, and ER contributed to the literature review, critically reviewed the manuscript, and gave comments. All authors agree to be accountable for the content of the work.

## Conflict of Interest

The authors declare that the research was conducted in the absence of any commercial or financial relationships that could be construed as a potential conflict of interest.
